# Personality traits, dieting self-efficacy and health behaviors in emerging adult women: implications for health promotion and education

**DOI:** 10.34172/hpp.2020.36

**Published:** 2020-07-12

**Authors:** Małgorzata Obara-Gołębiowska, Justyna Michałek-Kwiecień

**Affiliations:** ^1^Department of Clinical Psychology and Psychology of Developement and Education, University of Warmia and Mazury, Olsztyn, Poland; ^2^Institute of Psychology, University of Gdańsk, Poland

**Keywords:** Personality, Self-efficacy, Health behavior, Female, Young Adult, Health promotion

## Abstract

**Background:** The purpose of this study was to explore the associations between personality traits and dieting self-efficacy as well as health behaviors among emerging adult women.

**Methods:** In this cross-sectional study, the sample consisted of 161 participants in age from 19 to 25 years, who were administered the NEO-Five-Factor Inventory, the Health BehaviorsInventory (HBI), and the Dieting Self-Efficacy Scale (DIET-SE).

**Results:** Our findings indicated that personality traits explained both health behaviors and dieting self-efficacy (F = 6.21, df = 5,155, P<0.001, F = 6.42, df = 5,155, P<0.001, respectively).Neuroticism (B = -0.45, P<0.01) and agreeableness (B = 0.39, P<0.01) were investigated as significant predictors of females’ health behaviors, whereas extraversion (B = -0.40, P<0.001),agreeableness (B = 0.20, P<0.05), and conscientiousness (B = 0.33, P<0.01) were related to dieting self-efficacy. In addition, the results suggested the mediational effect of dieting self efficacy in the relationship between personality traits (i.e., consciousness and agreeableness)and general health behaviors.

**Conclusion:** The role of personality traits for dieting self-efficacy as well as physical health among emerging adult women was confirmed. As dieting self-efficacy turned out to be a mediation factor, the findings may be used in psychoeducation for patients.

## Introduction

Human health behaviors significantly determine the prevalence of the most common civilization diseases in the 21st century, e.g., cardiovascular disease, cancer or obesity.^1,2^ Recent studies have indicated that health behaviors, including nutritional behaviors, depend not only on the social environment, but also on human personality characteristics.^3-6^ Moreover, a review of the literature has emphasized that personality traits play an important role in maintaining and restoring health.^7-14^

In studies of personality and health behaviors, the five-factor personality model is currently the most widely-used and it includes the following dimensions: 1. neuroticism, 2. extraversion, 3. openness to experience, 4. agreeableness, 5. conscientiousness.^15^ Studies have indicated that high neuroticism positively affects alcohol consumption and cigarette smoking,^10-12^ whereas it is negatively correlated with physical activity.^13,14^ In turn, high extraversion is associated with greater physical activity but also with more frequent consumption of alcohol and smoking.^10-14^ Openness is positively related to consumption of fruits and vegetables, physical activity, as well as alcohol use and smoking.^9-14^ However, many studies have shown no significant relationship between openness and prospective health outcomes or changes in health.^16-19^ It has also been noted that higher agreeableness affects less frequent consumption of alcohol and cigarettes.^7^ Conscientiousness is positively correlated with dietary behavior,^20^ physical activity, and lower consumption of alcohol or cigarette smoking.^7^ Therefore, the associations between personality traits as defined in the five-factor model and health behaviors have been well documented. Considering health behaviors, recent research has indicated that dieting self-efficacy is one of the most important factor determining the return to normal eating habits. Recent studies focusing on the process of weight reduction have suggested that self-efficacious individuals believe they can overcome dietary challenges and obstacles. Therefore, they show more effort and stronger persistence in adhering to their slimming related goals.^21^ Such people also present better coping strategies because of the initiation of behavioral change and learning from their experience how to better manage difficult situations.^22-24^ Furthermore, self-efficacious individuals after breaking their diets recover more quickly and better adhere to their previous goals. To conclude, dieting self-efficacy is a construct related to eating behaviors, which are one of the most important factors influencing healthy lifestyles.

In addition, recent studies have emphasized that an important time for disease prevention and health promotion, including eating habits, is the transition from adolescence to adulthood.^25^ Emerging adulthood defined as a stage of life between adolescence and late twenties (usually defined as 18–25 years of age), which is linked with the transition to independent living, intimate relationships or parenthood.^26,27^ Research has suggested that this life phase can be a critical time for establishing independence and adopting lasting health behavior patterns. In addition, unhealthy lifestyle characteristics during emerging adulthood can be associated with increased risk for chronic disease.^25^ However, more work is needed to explore how the unique characteristics of emerging adulthood, e.g., personality traits, may contribute to establishing behavioral patterns and the possible vulnerability of this life stage to various influences.

### The current study

Despite the existence of studies showing the influence of psychological determinants on a person’s lifestyle, it is not fully known how personality is associated both with dieting self-efficacy and health behaviors. In addition, personality traits are rather stable dispositions, while dieting self-efficacy may be modified through intervention.^28^ In general, perceived self-efficacy exemplifies the belief that one can change risky health behaviours by own activity, and it has become extensively applied in a paradigm of addiction and relapse.^29-31^ Based on the literature review, it is known that motivational changes in one health domain may impact motivational changes in other health domains.^32-34^ The phenomenon of generalization of behavior change from one sphere to another has been described in research on cognitive and behavioral psychology.^35^

In this context, further research is needed to explore the determinants of health behaviors, especially in order to design effective health promotion and intervention programs.^36^ We examined these associations in a non-clinical sample of emerging adult women attending university, as research has confirmed their heightened risk for disordered eating behavior and experiencing body image disturbance.^37,38^ Thus, the current study is aimed at: 1) verifying the possibility of generalizing self-efficacy from dieting effectiveness on general health behaviors; 2) assessing the relationship between personality traits and dieting self-efficacy as well as health behaviors among emerging adult women; 3) examining the mediating effects of dieting self-efficacy between personality traits and health behaviors.

## Materials and Methods

### Participants and procedure

This cross-sectional study involved 161 women aged 19-25 years (M = 19.52, SD = 0.96), mean body mass index (BMI) = 21.64 (SD = 3.07), primary care patients from Warminsko-Mazurskie province. The study took place between January and June 2016. The rolling snowball (referral) sampling method was used. These were an absence of mental illness, chronic metabolic diseases, pregnancy and lactation as well as agreeing to participate in the study.Most of the respondents had secondary education, were city dwellers and were unmarried.

The questionnaires were filled in after the study aim had been explained by suitably qualified staff. All participants gave their written consent to participate in the study and completed printed self-report questionnaires. Participation was anonymous, and no payment was offered to the participants.

### Measures 

*Personality*

The NEO-FFI Costa and McCrae Personality Inventory^15^ in the Polish adaptation of Zawadzki et al^39^ was used to measure personality traits. This questionnaire provides information about five personality dimensions based on the Big Five model, i.e., neuroticism, extraversion, openness to experience, agreeableness and conscientiousness. Neuroticism determines the level of emotional balance. Extraversion reflects the quality and number of social interactions, as well as the level of activity, energy and ability to feel positive emotions. Openness to experience is characterized by the individual’s tendency to seek life experiences, tolerance for novelty and cognitive curiosity. Agreeableness determines positive or negative attitudes towards others. In turn, conscientiousness describes the degree of organization, perseverance and motivation of an individual in fulfilling his or her goals of life.^39^ Internal consistency reliability coefficients (Cronbach’s α) for the NEO-FFI subscales in the sample were .73 for neuroticism, 0.70 for extraversion, 0.55 for openness to experience, 0.75 for agreeableness, and .78 for conscientiousness. The respondent answers on a 5-point Likert scale (1. describes me incorrectly; 5. describes me correctly). Subscales are scored by summing the values of items in each dimension. The higher the score obtained by the respondent, the higher level of each personality dimension.

*Health behavior*

The Health Behaviors Inventory (HBI)^40^ was used to measure health behaviors. The HBI consists of 24 items and assesses general health-promoting behaviors and the severity of four categories of health behaviors: correct eating habits that determine the type of food being eaten; preventive behaviors regarding compliance with health recommendations; health practices covering daily habits of recreation, sleep and physical activity; and positive mental attitudes associated with avoiding negative emotions and strong stress (the Cronbach’s α coefficient for the overall scale was 0.79). The respondent answers on a 5-point Likert scale (1- almost never; 2- rarely; 3- from time to time; 4- often; 5- almost always) and the possible score ranged from 24-120. The higher the score obtained by the respondent, the higher the level of severity of health behaviors.

*Dieting self-efficacy*

The Dieting Self-Efficacy Scale (DIET-SE) by Stich et al^41^ in the Polish adaptation of Obara-Gołębiowska and Michałek-Kwiecień^42^ was used in the study. The DIET-SE consists of 11 items. It asks respondents to indicate their confidence in their ability to resist a variety of eating temptations. The DIET-SE contains three subscales. First subscale, HCF (high–caloric food), describes situations of the exposure to high-caloric food as an obstacle in dieting. The second, SIF (social and internal factors), presents situations in which social (e.g., dinner with family) or internal factors (e.g. being tired) can also make it difficult to resist the food temptation. The third subscale (negative emotional events, NEE) shows examples where emotional discomfort can be the cause of unplanned eating. In the present study, the internal consistency analysis showed high internal consistency for the overall scale (11 items) (α = 0.79) [for subscales: HCF (4 items), α = 0.66; SIF (4 items), α = 0.63 and NEE (3 items), α = 0.65]. The respondent answers on a 5-point Likert scale - yes (1 point); 2. Rather yes (2 points); 3. Rather not (3 points); 4. No (4 points). Item sums defined subscale scores and the total score. The higher the score obtained by the respondent, the higher level of each DIET-SE subscale.

### Statistical analysis

The IBM SPSS statistics software (version 23.0) (IBM SPSS Statistics, IBM Corp, Armonk, USA) was used to compute Pearson’s correlation coefficients to examine associations between variables. Two linear regressions analyses were performed to explain general health behaviors and dieting self-efficacy respectively, in both using all personality traits as independent variables. The *P* values less than 0.05 were considered statistically significant. Finally, to test if the impact of females’ personality traits on general health behaviors is mediated by dieting self-efficacy, mediation analyses based on the PROCESS bootstrapping macro^43^ were conducted.

## Results

The characteristics of the participants are summarized in Table 1.

The Pearson’s correlation coefficients among variables are displayed in Table 2. As expected, neuroticism was significantly negatively associated with the general index of intensity of health behaviors. Moreover, this personality trait was negatively correlated with only one subscale of diet self-efficacy – the negative emotional events dimension. Extraversion was positively related to the general index of intensity of health behaviors. In addition, the level of extraversion was negatively correlated with the social and internal factors dimension of dieting self-efficacy. No significant correlations were found between the level of openness to experience and the general index of intensity of health behaviors as well as diet self-efficacy. This result was consistent with prior research. According to our results, the level of agreeableness was positively associated with the general index of intensity of health behaviors. The level of this trait was also positively related to dieting self-efficacy and its two dimensions, i.e., social and internal factors, as well as negative emotional events. As expected, the significant correlations between conscientiousness and both general health behaviors and dieting self-efficacy were most frequent. Specifically, the level of this trait was positively associated with the general index of intensity of health behaviors. Moreover, conscientiousness was significantly related to dieting self-efficacy and all its dimensions, i.e., high-caloric food, social and internal factors, as well as negative emotional events.

As can be seen in Table 2, in accordance with previous predictions, positive correlations were found between general health behaviors and dieting self-efficacy as well as its subscales. Specifically, the general index of intensity of health behaviors (HBI) was positively related to DIET-SE, SIF, and NEE.

Two multiple linear regression analyses were carried out to examine the associations between personality traits and dieting self-efficacy as well as general health behaviors (Table 3). In the first linear regression analysis, the model was significant and explained 16% of the variance of dieting self-efficacy (*F* = 6.42, *df* = 5,155, *P* < 0.001). Dieting self-efficacy was significantly predicted by extraversion (*β* = -0.31, *P* < 0.001), agreeableness (*β* = 0.18, *P* < 0.05) and conscientiousness (*β* = 0.28, *P* < 0.01). The negative regression coefficient for extraversion indicated that lower level of extraversion was associated with higher level of dieting self-efficacy, whereas the positive regression coefficients of the other two predictors showed that higher levels of agreeableness as well as conscientiousness were associated with higher level of dieting self-efficacy.

The general index of intensity of health behaviors was also significantly predicted by personality traits in total. The model explained 14% of the variance of general health behaviors (*F* = 6.21, *df* = 5,155, *P* < 0.001). However, only neuroticism and agreeableness were identified as significant predictors of general health behaviors (*β* = -0.27, *P* < 0.01, *β* = 0.22, *P* < 0.01, respectively). Thus, lower level of neuroticism and higher level of agreeableness were associated with more healthy behaviors.

In the next step, we conducted the mediation analyses of indirect effect of dieting self-efficacy in relation between personality traits (model I for neuroticism, model II – extraversion, model III – openness to experience, model IV – agreeableness, model V – conscientiousness) and the general index of health behaviors. The obtained results indicated that only two models, i.e., models IV and V, are significant [*R*^2^ for the IV model = 0.17, *F* (2, 166) = 16.884, *P* < 0.001; *R*^2^ for the V model = 0.15, *F* (2, 166) = 14.456, *P* < 0.001].

**Figure 1:**
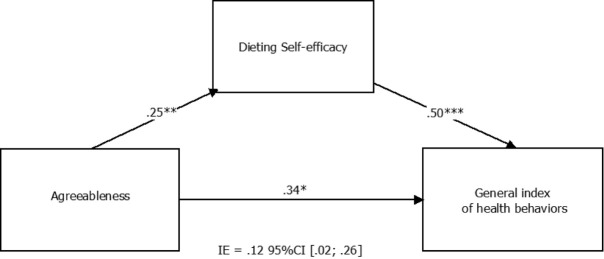
Mediation model for relationship between females’ agreeableness and general index of health behaviors with dieting self-efficacy as a mediator.
*Note* . The indirect effect (IE) is statistically significant at the 95% confidence interval (CI) when the CI does not include zero.

The mediation analysis indicated that the effect of females agreeableness on general health behaviors was partially mediated by dieting self-efficacy; the bootstrap confidence interval for the indirect effect (*b* = 0.12) based on 5000 bootstrap samples was entirely above zero (95% CI: 0.017, 0.262). The direct effect of females agreeableness on general health behaviors was significant (*b* = 0.34, SE= 0.12, *t* = 2.59, *P* < 0.05, 95% CI for *b* : 0.08, 0.59) (see Figure 1).

**Figure 2:**
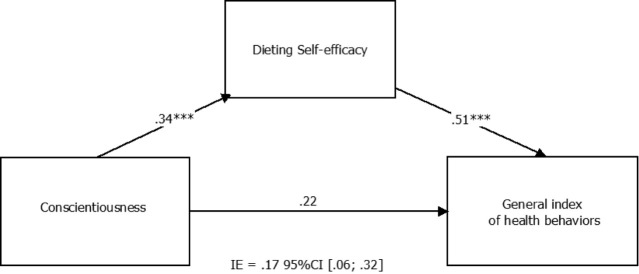
Mediation model for relationship between females’ conscientiousness and general index of health behaviors with dieting self-efficacy as a mediator.
*Note* . The indirect effect (IE) is statistically significant at the 95% confidence interval (CI) when the CI does not include zero.

In the model V, dieting self-efficacy was found to be a significant mediator (*b* = 0.17, 95% CI: 0.059, 0.324) between conscientiousness and health behaviors (see Figure 2). The direct effect of females conscientiousness on general health behaviors was not significant (*b* = 0.22, SE = 0.14, *t* = 1.59, *P* > 0.05, 95% CI for *b* : -0.052, 0.485).

## Discussion

The above presented results have been consistent with expectations and emphasized the role of personality traits for health behaviors among emerging adult women.^44-46^ In the current study, neuroticism was significantly negatively associated with general health behaviors as well as with one of the dimension of the dieting self-efficacy, i.e., negative emotional events. Previous studies also indicated that neuroticism is associated with a higher risk of developing disease.^47^ Apart from predicting future self-rated health^16,19,48^ and decline in self-rated health,^49,50^ neuroticism also acts as an important prospective risk factor when an individual’s health is rated by a physician rather than being self-reported.^51^ Studies on personality traits of obese people have confirmed their significantly higher level of neuroticism,^52^ which may be associated with emotional overeating.^53^ To conclude, according to studies high neuroticism plays an important role as a risk factor for poor health.

In our study, as expected, extraversion was positively related to health behaviors. It seems that extraversion can be a protective factor considering health outcomes, i.e., higher scores and increases in extraversion over time are associated with better prospective self-rated health.^16,19^ However, extraversion can be also related to risky health behaviors. It is known that extraversion (its facet of activity) is positively associated with the level of physical exercise which is in accordance with a healthy lifestyle.^13^ On the other hand, the high level of sensation seeking facet of extraversion is linked to increased alcohol consumption,^54,55^ cigarette smoking,^56^ risky driving behaviors^57,58^ and risky sexual behaviors.^59^ In the current study, extraversion was also negatively correlated with the social and internal dimension of dieting self-efficacy. The high level of extraversion, particularly sociability, promotes eating during social gatherings. For this reason, extraversion was significantly elevated in obese individuals compared to those with normal body mass.^60^ Thus, extraversion may play the positive as well as negative role in preventive and risky health behaviors.

As expected, no significant correlations were found between the level of openness to experience and health behaviors as well as dieting self-efficacy. Considering openness to experience, many studies did not find any significant relationship between this dimension of personality and prospective health outcomes or health changes.^16-19^

Our findings revealed that agreeableness is positively associated with health behaviors and dieting self-efficacy. It is consistent with the results from previous studies showing the importance of this personality trait in health outcomes. For example, it has been found that lower agreeableness is associated with faster accumulation of morbidity, over time.^51^ Therefore, the protective role of agreeableness in health behaviors was confirmed.

Regarding conscientiousness, as predicted, we found significant associations between the level of this trait and health behaviors as well as dieting self-efficacy. Specifically, higher conscientiousness turned out to be related to a lower prospective risk of disease and to lower burden of disease.^48^ In addition, it was found that higher conscientiousness predicts lower physician-assessed illness burden accumulation over time.^50,51^ Individuals with higher level of conscientiousness were found to report better subsequent self-rated health.^16,19,48,50^ According to longitudinal and meta-analytic studies, this personality trait seems to be a protective factor against the development of overweight and eating disorders.^61-63^

Despite the significant relationship between the above five dimensions of personality and health behaviors, according tothe multiple linear regression analysis the most important personality factors turned out to be neuroticism, agreeableness and conscientiousness. Specifically, neuroticism and agreeableness were identified as significant predictors of general health behaviors.The multiple linear regression analysis also showed that dieting self-efficacy was significantly predicted by extraversion, agreeableness, and conscientiousness. Thus, lower levels of extraversion, higher levels of agreeableness and conscientiousness were associated with higher levels of dieting self-efficacy.

Additionally, we analyzed the relationship between intensity of health behaviors and dieting self-efficacy. As we expected, general health behaviors were positively related to dieting self-efficacy and its dimensions, i.e., social and internal factors, and negative emotional events. Therefore, dieting self-efficacy seems to be in accordance with a general healthy lifestyle.^41^

Moreover, it is worth to emphasize that our results suggested the mediational effect (partial or full) of dieting self-efficacy in the relationship between personality traits (i.e., consciousness and agreeableness) and the general index of health behaviors. Health behaviors in women with low levels of agreeableness and conscientiousness could be improved by strengthening their dieting self-efficacy.^28^

The major limitation of the present study is that there was no data available on physical diseases of the participants. Replication of these findings with the use of various disease markers and medical information would be needed. Particularly, future research should focus on the effects of personality traits and dieting self-efficacy on changing health behaviors.

## Conclusion

In conclusion, our research confirmed the relationship between personality traits and health behaviors among emerging adult women. In addition, dieting self-efficacy turned out to be a mediator of this relationship. Knowledge obtained from the current study may be used in psychoeducation for emerging adult women. The conclusion from the present study may also be used in the psychotherapy of somatic patients. Moreover, for the correct diagnosis and treatment of observed disorders, it is important to expand patients’ awareness of factors that are associated with their health behaviors. These findings also can be useful in planning and guiding programs in preventive medicine. It is known that treatment of personality disorders^64^ with the use of personality profile^65^could also help to improve functioning and minimize risk exposure to health-threatening behaviors. We believe that incorporating personality factors into public health policy offers many benefits at almost no cost.^44^

## Ethical approval

The study was approved by the Bioethics Committee of University of Warmia and Mazury in Olsztyn, Poland.

## Competing interests

No potential conflict of interest was reported by the authors.

## Funding

This research was supported by the University of Warmia and Mazury in Olsztyn, Poland.

## Authors’ contributions

MOG and JMK conceptualized the study. MOG collected the data. JMK analyzed the data. MOG and JMK contributed to writing and revising the manuscript.

## Acknowledgments

We gratefully thank the participants who took part in the current study.

**Table 1:** Summary of demographic characteristics

**Characteristic**	**No. (%)**
Inhabitancy	
Town >100 000	97 (60)
Town <100 000	48 (30)
Village	16 (10)
Education	
Secondary	113 (70)
Higher	48 (30)
Marital status	
Single	129 (80)
Married	32 (20)

**Table 2:** Correlations among study variables

**Variable**		**1.**	**2.**	**3.**	**4.**	**5.**	**6.**	**7.**	**8.**	**9.**	**10.**
1.	Neuroticism	–									
2.	Extraversion	-0.34**	–								
3.	Openness	<0.01	0.14	–							
4.	Agreeableness	-0.14	0.20**	-0.13	–						
5.	Conscientiousness	-0.24**	0.33**	0.07	0.32***	–					
6.	General index of intensity of health behaviors	-0.33***	0.19*	0.02	0.29***	0.20*	–				
7.	Dieting self-efficacy scale	-0.13	-0.13	<-0.01	0.22**	0.27**	0.33***	–			
8.	High –Caloric Food	-0.01	-0.10	<0.01	0.08	0.18*	0.15	0.32***	–		
9.	Social and Internal factors	-0.08	-0.19*	0.03	0.18*	0.22**	0.30***	0.39***	0.17*	–	
10.	Negative emotional events	-0.23**	0.01	-0.04	0.26**	0.24**	0.33***	0.31***	0.25**	0.28***	–

*Note* . n = 161, * *P* < 0.05, ** *P* < 0.01, *** *P* < 0.001.

**Table 3:** Multiple regression analysis for variables predicting general index of intensity of health behaviors

**Variabl**e	**Dieting Self-Efficacy**					**General Index of Intensity of Health Behaviors**				
	**B**	**SE(B)**	**β**	**t**	***P*** **value**	**B**	**SE(B)**	**β**	**t**	***P*** **value**
Neuroticism	-0.15	0.08	-0.14	-1.82	0.071	-0.45	0.13	-0.27	-3.47	0.001
Extraversion	-0.40	0.11	-0.31	-3.78	0.000	0.05	0.16	0.03	0.33	0.745
Openness	0.07	0.10	0.05	0.63	0.528	0.09	0.16	0.04	0.54	0.587
Agreeableness	0.20	0.09	0.18	2.25	0.026	0.39	0.14	0.22	2.82	0.005
Conscientiousness	0.33	0.10	0.28	3.44	0.001	0.09	0.15	0.05	0.62	0.535
*R* ^ 2 ^	0.17					0.17				
Adjusted *R*^2^	0.16					0.14				
*F* (df1,df2)	6.42 (5,155)***					6.21 (5,155)***				

*Note* . * *P* < 0.05, ** *P* < 0.01, *** *P* < 0.001.
